# Efficacy of an internet-based cognitive behavioral therapy for subthreshold depression among Chinese adults: a randomized controlled trial

**DOI:** 10.1017/S0033291722000599

**Published:** 2023-07

**Authors:** Yuchen Ying, Yunxin Ji, Fanqian Kong, Minyao Wang, Qiqi Chen, Li Wang, Yanbin Hou, Libo Yu, Lijie Zhu, Pingping Miao, Jing Zhou, Li Zhang, Yiling Yang, Guanjun Wang, Ruijia Chen, Denong Liu, Wenjun Huang, Yueer Lv, Zhongze Lou, Liemin Ruan

**Affiliations:** 1 Department of Psychosomatic Medicine, Ningbo First Hospital, Ningbo Hospital of Zhejiang University, Ningbo, Zhejiang, People's Republic of China; 2 Department of Elderly Health Care and Management, School of Health Services and Management, Ningbo College of Health Sciences, Ningbo, Zhejiang, People's Republic of China; 3 Department of Medical Record and Statistics, Ningbo Medical Center Lihuili Hospital, Ningbo, Zhejiang, People's Republic of China; 4 School of Medicine, Ningbo University, Ningbo, Zhejiang, People's Republic of China; 5 Department of Statistics and Programming, Jiangsu Hengrui Pharmaceuticals Co. Ltd, Shanghai, People's Republic of China; 6 Central Laboratory of the Medical Research Center, Ningbo First Hospital, Ningbo, Zhejiang, People's Republic of China

**Keywords:** Chinese populations, internet-based cognitive-behavioral therapy, randomized controlled trial, subthreshold depression

## Abstract

**Background:**

Subthreshold depression (sD) negatively impacts well-being and psychosocial function and is more prevalent compared with major depressive disorder (MDD). However, as adults with sD are less likely to seek face-to-face intervention, internet-based cognitive-behavioral therapy (ICBT) may overcome barriers of accessibility to psychotherapy. Although several trials explored the efficacy of ICBT for sD, the results remain inconsistent. This study evaluated whether ICBT is effective in reducing depressive symptoms among Chinese adults with sD.

**Methods:**

A randomized controlled trial was performed. The participants were randomly assigned to 5 weeks of ICBT, group-based face-to-face cognitive-behavioral therapy (CBT), or a waiting list (WL). Assessments were conducted at baseline, post-intervention and at a 6-month follow-up. The primary outcome measured depressive symptoms using the Center for Epidemiological Studies Depression Scale (CES-D). Outcomes were analyzed using a mixed-effects model to assess the effects of ICBT.

**Results:**

ICBT participants reported greater reductions on all the outcomes compared to the WL group at post-intervention. The ICBT group showed larger improvement on the Patient Health Questionnaire-9 (PHQ-9) at post-intervention (*d* = 0.12) and at follow-up (*d* = 0.10), and with CES-D at post-intervention (*d* = 0.06), compared to the CBT group.

**Conclusions:**

ICBT is effective in reducing depressive symptoms among Chinese adults with sD, and improvements in outcomes were sustained at a 6-month follow-up. Considering the low rates of face-to-face psychotherapy, our findings highlight the considerable potential and implications for the Chinese government to promote the use of ICBT for sD in China.

## Introduction

Subthreshold depression (sD), although the definition is too broad to be used, refers to having a higher score than a certain cut-off in self-rated depression scales or having 1–2 significant depressive symptoms; however, it fails to meet the Diagnostic and Statistical Manual of Mental Disorders (DSM) criteria for major depression (Cuijpers et al., 2014). Compared with diagnosed clinical depression, subthreshold forms of depression are more prevalent (Cuijpers, Huibers, Ebert, Koole, & Andersson, 2013), negatively impact subjective well-being and psychosocial function (Cuijpers & Smit, 2004), are related to increased use of health care services (Goldney, Fisher, Dal Grande, & Taylor, 2004) and immense economic costs (Cuijpers et al., 2007). Therefore, it is necessary to develop appropriate interventions to treat sD.

Internet-based cognitive-behavioral therapy (ICBT) is an online intervention that can overcome some limitations of face-to-face cognitive-behavioral therapy (CBT; Andersson, Titov, Dear, Rozental, & Carlbring, 2019). Compared to traditional CBT, ICBT can be conducted anytime and anywhere with the internet, offers easy access to qualified therapists, relatively lower-costs, and provides privacy protection for patients who fear stigmatization. ICBT is therefore suitable for people with sD who are less likely to seek professional assistance (Zhou, Li, Pei, Gao, & Kong, 2016) and face-to-face intervention (Cuijpers, van Straten, Warmerdam, & van Rooy, 2010).

Although several randomized controlled trials (RCT) explored ICBT's efficacy for sD, the results, especially regarding evidence on the long-term efficacy, remain inconsistent; some RCTs showed the superiority of ICBT over control conditions at follow-up, while others did not (Reins et al., 2021; Zhou et al., 2016). Thus, the present study aims to clarify the long-term efficacy of ICBT on sD. Furthermore, an open research question is whether ICBT is as effective as face-to-face CBT in people with sD. Although preliminary evidence shows that guided ICBT could produce equivalent overall effects for depression as regular face-to-face CBT (Andersson, Topooco, Havik, & Nordgreen, 2016), only one study found that ICBT is more effective for sD (Spek et al., 2008). Furthermore, as face-to-face psychotherapy is less effective for sD than for major depression (Cuijpers et al., 2014), and people with mild depressive symptoms are more likely to seek less intensive treatment like ICBT (Zhou et al., 2016), it is important to compare the efficacy between ICBT and face-to-face CBT for sD.

Major depressive disorder (MDD) was the most common mood disorder in China with a lifetime prevalence of 3.4% and 12-month prevalence of 2.1%; considering China's 1.4 billion population, which indicates that an exceptionally large number of people are affected by MDD (Huang et al., 2019). Almost all individuals with MDD are assumed to initially pass through a period of sD (Frank et al., 1991), and studies showed that the incidence of sD among Chinese adolescents has reached 30~40% (Liu, Zeng, & Li, 2016). However, as Chinese people are influenced by cultural values, most people are unwilling to seek face-to-face psychotherapy, despite having clinical depressive symptoms (Ren, Li, & Zhao, 2016); thus, the treatment rates for depression are very low in China (Huang et al., 2019). To resolve this issue, China released the Action Plan for Depression Prevention and Treatment in September 2020 (Xinhua News Agency, 2020) to improve access to online interventions (e.g. ICBT) for depression. Nevertheless, although the efficacy of ICBT for sD has been examined among adults in western countries (Reins et al., 2021; Zhou et al., 2016), limited studies have focused on the Chinese population, and ICBT is rarely used in China (Yao, Chen, & Xu, 2020). Therefore, evidence-based research is needed to promote the use of ICBT for sD in China.

This study aimed to test the following hypotheses: (1) ICBT participants would demonstrate significant improvements in the measures of depressive symptom and other secondary outcomes when compared with control participants, and these improvements would be sustained at a 6-months follow-up; (2) there would be no significant difference between internet-based and face-to-face CBT for sD on the reduction of depressive symptoms and other secondary outcomes; and (3) the use of ICBT would be effective and acceptable for Chinese adults with sD.

## Method

### Study design

This study conducted a 3-group RCT to explore the efficacy of a clinician-guided ICBT compared with a group-based face-to-face CBT and a waiting list (WL) control group on sD. Study outcomes were assessed at baseline, immediately after the intervention, and at 6-month follow-up. However, there were only two assessment points for the WL group (i.e. baseline and post-intervention). Thus, there were no comparisons between the intervention groups and the WL group at 6-month follow-up. This trial received approval from the Medical Ethics Committee of Ningbo University (approval number: NBU-2020-139) and was pre-registered (registration number: ChiCTR2100049671).

### Participants and procedure

**Fig. 1.**
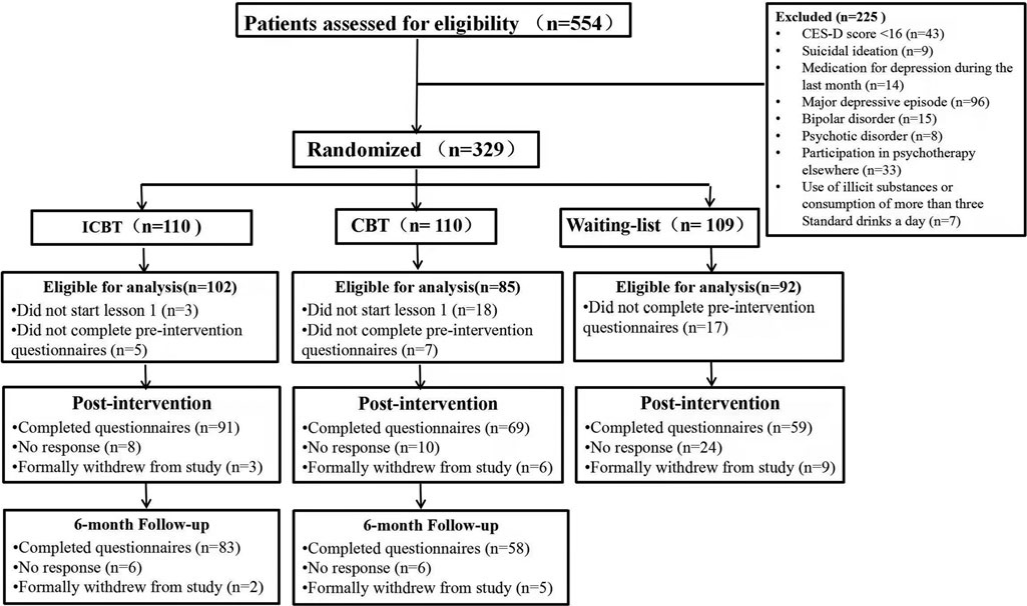
Flowchart of participants.

Overall, 554 participants were consecutively recruited between April and December 2020 from the general population via WeChat public account and related websites. Among them, 329 met the study's inclusion criteria. Figure 1 depicts the details of participant selection.

Participants had to meet the following inclusion criteria: (1) be a Chinese resident, (2) have a smart device and internet access, (3) be 18 years or older, (4) obtain a score of ⩾16 on the Center for Epidemiological Studies Depression Scale (CES-D), indicating sD (Buntrock et al., 2015), (5) have no suicidal ideation (indicated by a score of <2 on question 9 on the Patient Health Questionnaire 9-item (PHQ-9; Kroenke, Spitzer, & Williams, 2001), and (6) not take any medication for depression during the last month. All interested individuals provided informed consent and completed the online screening questionnaire.

Potentially eligible participants were invited for the telephone-administered Semi-Structured Clinical Interview for DSM (Fourth Edition) (*DSM-IV*) Axis Disorders (SCID) to assess final eligibility. The exclusion criteria were: (1) meeting the *DSM-IV* criteria for a major depressive episode (MDE), bipolar disorder, or psychotic disorder, (2) Current or past (in the past month) participation in psychotherapy elsewhere, and (3) using illicit substances or consuming >3 standard drinks daily. Eligible participants were administered standardized questionnaires, including all measures, and provided their sociodemographic data. Participants were thereafter randomly assigned to the following groups: ICBT group (*n* = 110), CBT group (*n* = 110), or WL group (*n* = 109), stratified by sex, age, and presenting depression symptom severity (symptom severity was determined using CES-D scores).

An independent research assistant completed randomization, according to a 1:1:1 ratio with a block size of six, using a computerized random number generator (www.random.org). Allocation was concealed from researchers using sequentially numbered, sealed opaque envelopes. The SCID was conducted by clinical psychologists, blind to the participants' assigned group. Participants could not be blinded to their allocation; however they were blinded to the condition of the WL group, as a control condition. Participants who completed post-intervention and follow-up questionnaires received ¥50 CNY (approximately $7.50 USD).

## Interventions

### Icbt

The *Healthy Psychological Station* comprised a 5-week-long clinician-guided ICBT delivered through a WeChat mini-program, which is tailored for the general Chinese population with depressive and anxiety symptoms. The program details are described elsewhere (Ying et al., 2021). In this trial, the *depression module* was used, which teaches practical CBT skills for depression and comprises five lessons with corresponding case stories and is based on the Chinese version of the Handbook of Cognitive-Behavioral Therapies (Dobson & Li, 2015). Table 1 depicts each lesson's descriptions. Participants were instructed to read the lessons in an individual format within 5 weeks, according to the same treatment timetable. However, they could arrange their specific study time during the week. The case stories were based on real cases of Chinese patients who had undergone CBT and recovered from sD or MDD. Clinicians who were blind to the hypothesis provided technical assistance and encouraged participants to use the program.

**Table 1.** Lesson content of the depression module

Lesson	Lesson content	Case story
Lesson 1	Getting support, building relationship, and signing contracts Introduction to listening, empathy, active attention, depression, CBT, and psychological education, etc. Understanding, relationship building, cognitive assessment (Preliminary establishment of case conceptualization and treatment plan)	Instructions and examples of building relationship and signing contracts with clinicians Instructions and examples of assessing own symptoms of depression Instructions and examples of establishing cognitive case conceptualization and treatment plan
Lesson 2	Introduction to the psychological meaning of symptoms of depression Introduction to the role of cognition (cognitive recognition, behavioral function analysis, cognitive behavior recording and reality test, etc.)	Instructions and examples of the cognitive recognition, behavioral function analysis, cognitive behavior recording and reality test
Lesson 3	Introduction to the relationship between cognition and emotion Introduction to the cognitive reconstruction and behavioral training (behavioral activation, and daily activity arrangement, etc.)	Instructions and examples of the cognitive reconstruction and behavioral training
Lesson 4	Introduction to the cognition, discussion, and reconstruction of basic beliefs Further introduction to behavioral training (further case conceptualization of cognition, vertical analysis of behavior, problem-solving strategies, further reconstruction of cognition and behavioral training, cognitive flash cards and cognitive motto, testing basic beliefs, etc.)	Instructions and examples of the reconstruction of basic beliefs and behavioral training
Lesson 5	Further introduction to the cognition, reconstruction of basic beliefs (consolidation of positive cognition, confirming and modifying basic beliefs, etc.) Further introduction to behavioral training (continued behavior training, etc.) Introduction to relapse prevention and constructing relapse prevention plans A summary of the five lessons and future behavior plan, etc.	Instructions and examples of the reconstruction of basic beliefs and behavior training Instructions and examples of relapse prevention and relapse prevention plans Instructions and examples of the summary and future plan

### Group-based face-to-face CBT

The group CBT protocol followed the same treatment components as the ICBT manual. There were 5 weekly sessions, each lasting 1–1.5 h. The interventions, delivered by two experienced licensed clinical psychologists who were trained in the approach and blind to the hypothesis, was conducted in a treatment room in Ningbo College of Health Sciences. Patients in the CBT group received the same treatment modules in the same chronological order as the ICBT group to ensure both groups received detailed information and treatment about depression (Andersson et al., 2013).

### Waiting list

Upon receiving the pre-intervention questionnaire, participants in the WL group were informed that the intervention would occur in a few weeks for them to enroll into the trial. There was no further contact or information provided. Following the post-intervention assessment, WL participants were debriefed about the study and provided CBT or ICBT access, according to their preferences.

## Measures

### Primary outcome measures

The Chinese version of the CES-D (Radloff, 1977), which showed good reliability and validity (Jiang et al., 2019; Lin, 1989), was used to assess depressive symptoms. The Cronbach's *α* was 0.90 in this study.

### Secondary outcome measures

The Chinese version of the Beck Depression Inventory-II (BDI-II; Beck, Steer, & Brown, 1996) and PHQ-9 (Kroenke et al. 2001), which showed good reliability and validity (Wang, Wang, & Xin, 2020; Wang et al., 2014), were used to assess depressive symptoms. The Cronbach's *α* in this study was 0.88 and 0.91 for the BDI-II and PHQ-9, respectively. The Chinese version of the Generalized Anxiety Disorder 7-Item (GAD-7; Spitzer, Kroenke, Williams, & Löwe, 2006), which showed good reliability and validity (Yu et al., 2018), was used to assess anxiety symptoms. The Cronbach's *α* was 0.87 in this study. The Chinese version of the Ruminative Response Scale (RRS; Nolen-Hoeksema and Morrow, 1991), which showed good reliability and validity (He et al., 2021), was used to assess rumination. The Cronbach's *α* was 0.76 in this study. Participants' satisfaction with and acceptability of the intervention were assessed after intervention using the following two questions, extracted from a similar study: (1) ‘Would you feel confident in recommending this program?’ and (2) ‘Was it worth your time doing the program?’ To these questions, participants responded with either ‘yes’ or ‘no’ (Dear et al., 2015).

## Statistical analysis

All analyses were conducted using SAS version 9.4 (SAS Institute, Cary, NC). A two-tailed *p* value <0.05 was considered statistically significant. The three groups' baseline characteristics were compared using either one-way analysis of variance (ANOVA) or Kruskal–Wallis tests for continuous measures and chi-square tests for categorical variables.

Participants in both ICBT and CBT groups who had not begun Lesson 1 were excluded from the analyses. The least-squares means (LS means) of outcomes were estimated, conducting an intention-to-treat (ITT) mixed-effects model for repeated measures (MMRM; Gueorguieva & Krystal, 2004) using a time × group interaction as an indicator of intervention effect. Furthermore, the effects were modeled using the restricted maximum likelihood (REML) estimation method with an unstructured (UN) covariance structure. Model fit was evaluated using Schwarz's Bayesian criterion. Appropriate contrasts in the MMRM were conducted by comparing mean changes for each group at each time-point and mean between-group differences.

Within-group and between-group effect size (Cohen's *d*) were based on the method suggested for mixed model analysis (Feingold, 2009; Morris & DeShon, 2002; Thorsell et al., 2011). Furthermore, confidence intervals (CIs) for the effect size were calculated using the method described by Kelley (Kelley, 2007). Effect sizes were categorized as small, medium, and large for Cohen's *d* > 0.2, *d* > 0.5, and *d* > 0.8, respectively (Cohen, 1988).

Finally, reliable change and a clinically significant improvement from baseline to post-intervention and 6-month follow-up were calculated for CES-D, according to the guidelines by Jacobson and Truax (Jacobson & Truax, 1991). The reliable change was defined as a drop of at least three points [using the criterion that Reliable Change Index (RCI) must be >1.96 and using the CES-D reliability (0.90) in the RCI formula] on the CES-D score, from baseline to post-intervention and follow-up, plus a CES-D post-intervention score <16 (defined as close to symptom-free status) to achieve clinically significant improvement. Chi-squared tests were used to evaluate the frequency differences in reliable change and in clinically significant improvements between the three groups. We also calculated the number needed to treat (NNT) and 95% CIs accordingly to achieve one additional reliable change and clinically significant improvement at post-intervention with WL as the reference group, respectively (Cook & Sackett, 1995).

## Results

### Participants' demographic characteristics

Table 2 outlines participants' demographic characteristics. There were no significant differences between the groups at baseline with respect to outcomes and demographic characteristics, indicating that randomization was successful. The participants' median age was 41.30 years (standard deviation (s.d.) = 13.35), 66.4% were women, and most participants were married (72.00%) and employed full-time (73.50%). Additionally, only 8.2% and 7.5% of the participants had an educational qualification higher than a certificate/diploma/university degree and a high-level income, respectively. Furthermore, nearly a quarter of the participants reported a history of MDE.

**Table 2.** Demographic characteristics of the participants

Participant characteristics	Total (*n* = 329)	CBT (*n* = 110)	ICBT (*n* = 110)	WL (*n* = 109)	*p*
Gender, *n* (%)					0.98
Male	90 (33.6)	28 (32.9)	31 (34.1)	31 (33.7)	
Female	178 (66.4)	57 (67.1)	60 (65.9)	61 (66.3)	
Age, Mean (s.d.)	41.3 (13.35)	43.7 (14.23)	39.9 (13.53)	40.4 (12.13)	0.13
Employment status, *n* (%)					0.90
Full-time job	197 (73.5)	65 (76.5)	66 (72.5)	66 (71.7)	
Part-time job	48 (17.9)	13 (15.3)	17 (18.7)	18 (19.6)	
Unemployed	11 (4.1)	3 (3.5)	4 (4.4)	4 (4.3)	
Student	12 (4.5)	4 (4.7)	4 (4.4)	4 (4.3)	
Marital status, *n* (%)					0.16
Unmarried	45 (16.8)	11 (12.9)	21 (23.1)	13 (14.1)	
Married	193 (72.0)	64 (75.3)	62 (68.1)	67 (72.8)	
Divorced	25 (9.3)	8 (9.4)	7 (7.7)	10 (10.9)	
Widowed	5 (1.9)	2 (2.4)	1 (1.1)	2 (2.2)	
Monthly income, *n* (%)					1.00
Low	43 (16.0)	12 (14.1)	16 (17.6)	15 (16.3)	
Medium	205 (76.5)	68 (80.0)	67 (73.6)	70 (76.1)	
High	20 (7.5)	5 (5.9)	8 (8.8)	7 (7.6)	
Education, *n* (%)					0.95
Primary school or below	9 (3.4)	3 (3.5)	3 (3.3)	3 (3.3)	
Junior high school	40 (14.9)	10 (11.8)	15 (16.5)	15 (16.3)	
Senior high school	197 (73.5)	67 (78.8)	65 (71.4)	65 (70.7)	
College or above	22 (8.2)	5 (5.9)	8 (8.8)	9 (9.8)	
History of MDE, *n* (%)	82 (24.9)	28 (25.5)	30 (27.3)	24 (22.0)	0.64

CBT, Cognitive-behavioral therapy; ICBT, Internet-based cognitive-behavioral therapy; WL, Waiting list; s.d., Standard deviation; MDE, Major depressive episode.

### Main outcomes

Table 3 depicts the LS means and s.d. for outcomes at baseline, post-intervention and at a 6-month follow-up for the three groups. The mixed-effects model analyses revealed significant time × group interactions on the CES-D (*F*_2140_ = 689.51, *p* < 0.001), BDI-II (*F*_2162_ = 112.42, *p* < 0.001), PHQ-9 (*F*_2156_ = 30.29, *p* < 0.001), GAD-7 (*F*_2132_ = 144.61, *p* < 0.001) and RRS (*F*_2152_ = 857.37, *p* < 0.001).

**Table 3.** Mean and standard deviation of outcomes for study conditions

Outcome	CBT (*n* = 110) Mean (s.d.)	ICBT (*n* = 110) Mean (s.d.)	WL (*n* = 109) Mean (s.d.)	Time × group	Significant contrasts between two groups
CES-D				*F*_2140_ = 689.51***	
Baseline	24.8 (4.70)	25.1 (2.47)	26.0 (1.38)		
Post-intervention	15.8 (2.66)	14.2 (4.67)	25.5 (1.18)		WL < ICBT*** WL < CBT*** CBT < ICBT**
6-month follow-up	15.7 (2.17)	14.8 (4.45)			
PHQ-9				*F*_2156_ = 30.29***	
Baseline	12.3 (2.36)	12.6 (2.40)	12.2 (2.58)		
Post-intervention	8.7 (2.74)	7.3 (2.12)	10.8 (2.34)		WL < ICBT*** WL < CBT*** CBT < ICBT***
6-month follow-up	8.6 (2.55)	7.5 (2.15)			CBT < ICBT*
BDI-II				*F*_2162_ = 112.42***	
Baseline	15.0 (2.30)	15.2 (2.11)	15.6 (2.16)		
Post-intervention	10.0 (2.37)	8.9 (2.92)	15.3 (1.92)		WL < ICBT*** WL < CBT***
6-month follow-up	9.7 (2.67)	9.5 (2.88)			
GAD-7				*F*_2132_ = 144.61***	
Baseline	13.1 (2.12)	12.9 (2.42)	13.0 (2.12)		
Post-intervention	8.6 (1.38)	8.3 (1.31)	12.7 (1.87)		WL < ICBT*** WL < CBT***
6-month follow-up	8.9 (0.90)	9.3 (2.26)			
RRS				*F*_2152_ = 857.37***	
Baseline	52.3 (3.64)	52.2 (3.90)	52.6 (7.84)		
Post-intervention	16.0 (1.25)	16.2 (1.60)	32.2 (3.81)		WL < ICBT*** WL < CBT***
6-month follow-up	16.6 (1.56)	16.7 (1.44)			

CBT, Cognitive-behavioral therapy; ICBT, Internet-based cognitive-behavioral therapy; WL, Waiting list; CES-D, Center for Epidemiologic Studies Depression Scale; BDI-II, Beck Depression Inventory-II; PHQ-9, Patient Health Questionnaire-9 Item; GAD-7, Generalized Anxiety Disorder 7-Item; RRS, Ruminative Response Scale.

*p < 0.05, **p < 0.005, ***p < 0.001.

Contrasts comparing change from baseline showed a consistent pattern of significant improvement at post-intervention and follow-up for both CBT and ICBT groups on all the outcomes (*p* < 0.001), whereas WL participants showed a significant increase on the PHQ-9 (*p* = 0.043) and RRS (*p* < 0.001).

Contrasts revealed that both CBT and ICBT resulted in a significantly larger reduction on all the outcomes compared with WL at post-intervention and follow-up (*p* < 0.001). The ICBT group showed significantly stronger improvement on the PHQ-9 at post-intervention (*p* < 0.001) and at follow-up (*p* = 0.015), and on the CES-D at post-intervention (*p* = 0.006) in comparison to the CBT group. No other significant differences between the CBT and ICBT groups were found.

## Effect size

### Primary outcome

Table 4 presents effect sizes (Cohen's *d*), calculated both within and between groups based on estimated means. The within-group effect sizes on the primary outcome measure CES-D were small for all treatments at post-intervention (CBT *d* = 0.23, 95% CI 0.20–0.26 *v.* ICBT *d* = 0.44, 95% CI 0.41–0.47) and follow-up (CBT *d* = 0.24, 95% CI 0.21–0.27 *v.* ICBT *d* = 0.47, 95% CI 0.44–0.49). The within-group effect size for CES-D for the WL group was 0.15 (95% CI 0.12–0.18) at post-intervention.

**Table 4.** Within and between-group effect sizes (Cohen's *d*) at post-intervention and at a 6-month follow-up

Outcome	Within-group effect size (95% CI)			Between-group effect size (95% CI)		
	CBT	ICBT	WL	ICBT *v.* CBT	CBT *v.* WL	ICBT *v.* WL
CES-D						
Post-intervention	0.23 (0.20–0.26)	0.44 (0.41–0.47)	0.15 (0.12–0.18)	0.06 (0.02–0.09)	1.15 (1.11–1.19)	0.48 (0.45–0.51)
6-month follow-up	0.24 (0.21–0.27)	0.47 (0.44–0.49)		0.04 (0.00–0.07)		
PHQ-9						
Post-intervention	0.30 (0.28–0.33)	0.49 (0.46–0.52)	0.12 (0.09–0.14)	0.12 (0.09–0.15)	0.16 (0.12–0.19)	0.35 (0.32 to −0.39)
6-month follow-up	0.34 (0.31–0.37)	0.46 (0.43–0.49)		0.10 (0.06–0.13)		
BDI-II						
Post-intervention	0.47 (0.44–0.49)	0.52 (0.49–0.54)	0.04 (0.01–0.06)	0.08 (0.05–0.11)	0.57 (0.53–0.60)	0.52 (0.48–0.55)
6-month follow-up	0.44 (0.41–0.47)	0.49 (0.46–0.52)		0.02 (−0.02 to 0.05)		
GAD-7						
Post-intervention	0.65 (0.62–0.68)	0.54 (0.52–0.57)	0.03 (0.00–0.06)	0.07 (0.03–0.10)	0.77 (0.73–0.80)	0.85 (0.81–0.89)
6-month follow-up	0.67 (0.64–0.70)	0.32 (0.29–0.35)		0.06 (−0.02 to −0.09)		
RRS						
Post-intervention	2.14 (2.09–2.18)	1.78 (1.74–1.82)	0.19 (0.16–0.22)	0.05 (0.02–0.08)	1.00 (0.97–1.04)	0.95 (0.91–0.99)
6-month follow-up	1.97 (1.93–2.01)	1.70 (1.66–1.73)		0.03 (0.01–0.07)		

CBT, Cognitive-behavioral therapy; ICBT, Internet-based cognitive-behavioral therapy; WL, Waiting list; CI, Confidence interval; CES-D, Center for Epidemiologic Studies Depression Scale; BDI-II, Beck Depression Inventory-II; PHQ-9, Patient Health Questionnaire-9 Item; GAD-7, Generalized Anxiety Disorder 7-Item; RRS, Ruminative Response Scale.

The between-group effect sizes for CES-D between the CBT group and ICBT group were small (post-intervention: *d* = 0.06, 95% CI 0.02–0.09; follow-up: *d* = 0.04, 95% CI 0.00–0.07), and between-group effect sizes for CES-D between the intervention groups and WL group ranged from small to large (CBT *v.* WL *d* = 1.15, 95% CI 1.11–1.19; ICBT *v.* WL *d* = 0.48, 95% CI 0.45–0.51).

### Secondary outcome

The within-group effect sizes for secondary outcomes ranged from small to large (post-intervention: CBT *d* range: 0.30–2.14; ICBT *d* range: 0.49–1.78; follow-up: CBT *d* range: 0.34–1.97; ICBT *d* range: 0.32–1.70) for the intervention groups. The between-group effect sizes for secondary outcomes were small (post-intervention: CBT *v.* ICBT *d* range: 0.05–0.12; follow-up: CBT *v.* ICBT *d* range: 0.02–0.10).

The within-group effect sizes for secondary outcomes were small (*d* range: 0.03–0.19), and the between-group effect sizes for secondary outcomes ranged from small to large (CBT *v.* WL *d* range: 0.16–1.00; ICBT *v.* WL *d* range: 0.35–0.95) for the WL group.

### Reliable change and clinically significant improvement

Table 5 shows the proportion of participants in each group who showed a reliable change or a clinically significant improvement for each time point. The three groups demonstrated significant differences post-intervention (*p* < 0.001), while there were no significant differences between the CBT and ICBT group at follow-up (*p* < 0.001). We calculated the NNT at post-intervention with the WL group as the reference. The NNT to achieve one additional clinically significant improvement were 3.04 (95% CI 2.23–4.36) and 2.62 (95% CI 2.18–3.35) respectively for CBT and ICBT. The NNT to achieve one additional reliable change were 1.86 (95% CI 1.52–2.39) and 1.79 (95% CI 1.45–2.33) respectively for CBT and ICBT.

**Table 5.** Proportion of participants showing clinically significant improvement or reliable change based on the Center for Epidemiologic Studies Depression Scale

Outcome	CBT *n* (%)	ICBT *n* (%)	WL *n* (%)	*χ*2	*P*	NNT (95% CI)	
						CBT *v.* WL	ICBT *v.* WL
Clinically significant improvement							
Post-intervention	28 (32.9)	39 (38.2)	–	49.002	<0.001	3.04 (2.23–4.36)	2.62 (2.18–3.35)
6-month follow-up	27 (31.8)	34 (33.3)	–	0.02	0.887		
Reliable change							
Post-intervention	55 (64.7)	68 (66.7)	10 (10.9)	69.229	<0.001	1.86 (1.52–2.39)	1.79 (1.45–2.33)
6-month follow-up	52 (61.2)	64 (62.7)	–	1.273	0.259		

CBT, Cognitive-behavioral therapy; ICBT, Internet-based cognitive-behavioral therapy; WL, Waiting list; NNT, Number needed to treat.

### Adherence, attrition, and response rates, and treatment satisfaction

The five lessons were completed by 83 of 102 ICBT participants (81.37%) and 55 of 85 CBT participants in 8 weeks (64.70%); the average number of lessons completed for ICBT, and CBT participants were 4.19 (s.d. = 3.35) and 3.76 (s.d. = 11.23), respectively. The mean total time spent with a clinical psychologist for each participant in the complete ICBT program was 31.08 min (s.d. = 21.94).

Furthermore, post-intervention data were collected from 91/102 (89.22%), 69/85 (81.18%), and 59/92 (64.13%) of ICBT, CBT, and WL participants, respectively. At a 6-month follow-up, data were collected from 83/102 (81.37%) and 58/85 (68.24%) of ICBT and CBT participants, respectively.

The ICBT and CBT participants who completed the post-intervention satisfaction questionnaires reported a high level of satisfaction. Further, 80 ICBT participants (87.91%) and 47 CBT participants (68.12%) felt confident in recommending the intervention to others; while 76 ICBT participants (83.52%) and 49 CBT participants (71.01%) reported that the intervention was worth doing.

## Discussion

### Principal findings

In this study, we examined the efficacy of clinician-guided ICBT on Chinese adults with sD, compared to a face-to-face CBT group and WL control group. We found that ICBT was effective from pre- to post-intervention and at a 6-month follow-up for depressive and anxiety symptoms, and rumination among adults with sD. In addition, over 30% and 60% of participants reported clinically significant improvement and reliable change for CES-D from pre-treatment to follow-up, respectively. Furthermore, 81.37% of participants completed the five lessons in the *Healthy Psychological Station,* and >80% of them reported a high degree of satisfaction. Overall, ICBT is effective in reducing sD in Chinese adults.

### Comparisons with previous work

The findings of the present study are encouraging and consistent with the results in previous studies concerning ICBT for sD (Cuijpers et al., 2014; Reins et al., 2021; Zhou et al., 2016). The between-group effect sizes for ICBT *v.* WL regarding reducing depressive symptom severity, was larger than that of two meta-analyses on ICBT for the treatment of sD (Reins et al., 2021; Zhou et al., 2016). This might be explained by the high heterogeneity in the meta-analysis with respect to target groups, intervention, and type of control group. When limited to those studies who used WL as the control group, the between-group effect sizes on depressive symptoms for ICBT at post-intervention and follow-up in this study correspond with the effect size of 0.71 (post-intervention) and 0.66 (3-month follow-up) found by Ebert et al. (2018) who did not use intensive guidance. Our findings were also consistent with the effect size of 0.46 found by Proudfoot et al. (2013) that used a complete self-help intervention; however participants received relatively intensive guidance in the present study (average 31.08 min). Although a previous study demonstrated that human support increases the effectiveness to eHealth interventions through accountability with an experienced coach (Mohr, Cuijpers, & Lehman, 2011), the question arises whether the lack of therapist guidance has a similar effect for sD in conditions that require fewer resources. Thus, more research is required to explore the relationship between therapist guidance and success in ICBT for sD.

Significant and higher improvement on the RRS were observed in the ICBT group, compared to the WL group. Previous findings indicated that ICBT was an effective treatment for repetitive, negative thinking among patients with mixed anxiety and depression (Newby, Williams, & Andrews, 2014), while the present study is the first, to our knowledge, to provide evidence on the effects of ICBT on reducing rumination among adults with sD. As rumination was considered a common proximal mechanism relating depressive risk factors to sD (Spasojević & Alloy, 2001), this important finding may demonstrate that depressive symptoms can be effectively reduced by relieving rumination through ICBT.

ICBT participants showed significant improvement on the CES-D and PHQ-9 with small effect size at post-intervention and showed significant improvement on the PHQ-9 with small effect size at follow-up, compared to CBT participants. Furthermore, a higher proportion of ICBT participants experienced reliable change and clinically significant improvement for CES-D at post-intervention and follow-up, which may be because of higher adherence found among ICBT participants than CBT participants in our study; this concurs with Spek *et al*.'s (2008) findings. Previous studies indicated that ICBT offers the highest benefits in individuals with positive attitudes (Schröder et al., 2018), while the relatively high attrition rate of the CBT group may decrease the effectiveness of face-to-face CBT (Cuijpers, Huibers, Ebert, Koole, & Andersson, 2013). There was a non-significant trend toward improvement on the other outcomes for CBT compared to ICBT except for CES-D and PHQ-9. This may be because our program design was developed based on conventional CBT, and ICBT participants received relatively intensive guidance, similar to face-to-face CBT and should thus have a similar treatment effect. However, no other study has provided a between-group effect size on the outcomes for ICBT *v.* CBT among adults with sD. Therefore, future research should use a larger sample to address this issue.

This study focuses on improving sD, and to the best of our knowledge, is the first study to support the feasibility and acceptability of ICBT for the Chinese population with sD. The results are encouraging and consistent with previous studies examining the use of ICBT for adults with sD in western countries (Reins et al., 2021; Zhou et al., 2016), while the adherence, response rates, and treatment satisfaction in this study were as high as or even higher than those observed in the aforementioned studies. There are three possible reasons for these findings. First, ICBT participants received relatively intensive therapist guidance, and due to the culture of obedience to the authority of doctors in China, therapist guidance may be more acceptable for Chinese adults compared to adults of other races or ethnic backgrounds, which can decrease participant attrition rate (Mohr et al., 2011). Second, the lessons and case stories of the program were developed in consultation with Chinese patients who had undergone CBT and recovered from sD or MDD; therefore, it was adapted culturally for the Chinese population. Third, for ICBT participants, an automated WeChat reminder was set to encourage adherence to the timetable. Furthermore, active outreach of tracing participants who missed scheduled attendance was conducted via telephone and email in both treatment arms.

Of the ICBT participants, 48.3% failed to achieve a reliable change at a 6-month follow-up. This may be because some ICBT participants reported that they experienced MDE. Researchers demonstrated that a history of MDE is one of the most common predictive factors for lack of treatment response (Andersson, Bergstrm, Hollndare, Ekselius, & Carlbring, 2004). However, lack of treatment response during follow-up may lead to various negative consequences. One possible side effect could be that participants with sD become less motivated for more intensive psychotherapy when the effects diminish after ICBT is completed (Zhou et al., 2016). Thus, future studies should evaluate the potential negative effects of ICBT for sD at long-term follow-up, especially in non-responders (Rozental et al., 2014).

### Implications

First, the effects on self-reported depression severity were almost moderate in size, which further supports the potential benefits of ICBT in treating depressive symptoms in the early stage. Studies using ICBT to prevent depression demonstrated that the treatment of sD may reduce the severity of symptoms and help prevent further deterioration and the onset of full-blown major depression (Buntrock et al., 2016). Second, the results suggested that guided ICBT can be as effective as face-to-face CBT for the treatment of sD. However, compared with face-to-face CBT, ICBT can be applied flexibly, conducted independently of time and place, and implemented with lower costs and threshold accessibility (Andersson et al., 2013). Third, this study adds to earlier studies by examining ICBT's feasibility and effectiveness for sD among the Chinese population and highlights the potential cost-effectiveness of ICBT interventions in China. Lu et al. (2021) found that the treatment rates of sD might be very low, and a few individuals receive adequate treatment in China, underscoring the importance of national programs to eliminate barriers to availability, accessibility, and acceptability of care for sD in China. Thus, promoting the use of ICBT in the management of sD for Chinese adults is recommended.

## Strengths and limitations

This study presents the following strengths. First, participants' mental health status was measured using validated measurement tools with good reliability and validity. Second, attrition rates were relatively low at post-intervention and at a 6-month follow-up; the linear mixed model provided an unbiased estimation of the ITT effect when it was assumed that participant data lost-at-follow-up were randomly missing; Third, the study's randomized controlled design with the direct comparison of two active conditions. Finally, we recruited participants from the general population rather than general practice or clinics, and thus, no biases were caused by individuals' help-seeking behavior and doctors' recognition of the disease (Paykel et al., 1997).

There are several limitations of our study. First, the results were based on participants' self-reported scores collected via the Internet, which could cause selection bias. Additionally, we relied on self-report measurements at post-intervention and follow-up and thus had no information regarding the diagnoses of depressive episodes during these stages. Second, most participants were better educated with higher income levels than the general Chinese population, which may help them learn more from ICBT (Imamura et al., 2014). Therefore, the generalization of the present findings may be limited. Moreover, we did not exclude participants with a history of MDE. Neither did we exclude participants who had depression treatment in the last month who may be on an improved trajectory. These may obfuscate the effects of the intervention (Andersson et al., 2004; Hadjistavropoulos, Pugh, Hesser, & Andersson, 2016). A further RCT should be conducted to test whether the ICBT program is effective in treating sD, using a larger sample with more diverse characteristics, without an MDE history and no treatment for depression over a longer period. Third, we did not monitor the additional use of other treatments during the trial period, which might have led to biased results. Fourth, choosing a group CBT intervention as a comparator group is a methodological limitation, which impacts patient engagement and participation in the therapeutic process. Future studies should be conducted using an individual CBT as a comparator. Finally, it is unclear whether the current results were maintained beyond the a 6-month follow-up period.

## Conclusions

This trial showed that ICBT is effective in reducing depressive symptoms among Chinese adults with sD, and the improvements in outcomes were sustained at a 6-month follow-up. Our study adds to existing literature indicating that ICBT, an intervention that requires less therapist time and may be more acceptable for adults with sD, may be a complement to face-to-face CBT in the treatment of sD. A large number of Chinese people are affected by sD, although the treatment rates of face-to-face psychotherapy for depression are low. Thus, the current study's findings further highlight the considerable potential of ICBT programs in improving online access to psychotherapy and producing reasonable symptom gains for these individuals, who may otherwise never receive mental health services.
